# Population-referenced multiregional brain-volume profiles associated with performance and concussion history in athletes: an exploratory study

**DOI:** 10.3389/fnhum.2026.1904810

**Published:** 2026-08-10

**Authors:** Mark Lindsay, Craig Harness, Owen Phillips, Kevin Aquino, Nathan Strong, Brady J. Williamson

**Affiliations:** 1 Department of Neuroscience, Queen's University, Kingston, ON, Canada; 2 Department of Anatomy, Queen's University, Kingston, ON, Canada; 3 BrainKey, Inc., San Francisco, CA, United States; 4 Department of Radiology, University of Cincinnati College of Medicine, Cincinnati, OH, United States

**Keywords:** brain volume, concussion, elite athletes, normative modeling, principal component analysis, sensorimotor performance, structural MRI

## Abstract

**Introduction:**

Elite athletes may exhibit structural brain profiles shaped by long-term training, selection into elite sport, exposure to repetitive head impacts, or combinations of these factors. Interpreting brain structure in this population is challenging because general-population reference values may not distinguish adaptive athlete-associated variation from injury-related or clinically meaningful change. In this exploratory cross-sectional study, we used age- and sex-referenced regional brain-volume percentiles as a population-referenced framework to examine whether multiregional structural profiles in elite athletes were associated with athletic performance, sensorimotor function, and reported concussion history.

**Methods:**

Fifty-one active and retired elite athletes underwent 3T structural MRI and robotic sensorimotor testing (KINARM). T1-weighted scans were segmented using BrainKey (brainkey.ai) to obtain age- and sex-referenced regional percentiles across 18 brain regions. Principal component analysis (PCA) was applied directly to the regional percentile measures, and Bayesian models assessed associations with ordered performance ratings, KINARM components, and reported concussion history.

**Results:**

Five principal components explained 71.4% of the variance. Brain PC1 was positively associated with higher performance-group membership [β = 0.53, 95% CrI (0.04, 1.02), posterior probability of direction = 0.983, *n* = 51]. Brain PC3 was positively associated with KINARM PC3 [β = 0.57, 95% CrI (0.01, 1.13), posterior probability of direction = 0.978, *n* = 44]. However, both components showed limited bootstrap stability, making this result highly exploratory. Reported concussion history was available for 50 participants and showed suggestive directional evidence for lower Brain PC2 [OR = 0.61, 95% CrI (0.36, 1.04), posterior probability of direction = 0.97] and higher Brain PC1 [OR = 1.57, 95% CrI (0.92, 2.71), posterior probability of direction = 0.95].

**Discussion:**

These exploratory findings indicate that population-referenced multiregional brain-volume profiles may be related to athletic performance, sensorimotor function, and reported concussion history. The results do not establish pathological or beneficial structural brain change and do not construct or validate an athlete-specific normative model. Instead, they identify candidate multiregional profiles for evaluation in larger, longitudinal athlete cohorts with matched non-athlete comparison groups.

## 1 Introduction

Elite athleticism can be considered a unique form of expertise. The neuroscientific importance of this population lies in the combination of prolonged, intensive motor practice and perceptual-cognitive demands. Thus, elite athletes provide a useful model for studying experience-dependent plasticity and the neural correlates of long-term motor expertise (Debarnot et al., 2014; Draganski et al., 2004; Gaser and Schlaug, 2003; Mann et al., 2007; May, 2011).

Given the physical demands of professional sport, especially in contact and collision sports such as football, hockey, and rugby, it is important to have reliable reference points for tracking health over time. One area in which this remains limited is brain health. Expertise in physical domains has been associated with reduced motor variability, altered neural recruitment during domain-relevant tasks, and structural or functional differences in systems supporting motor control and learning (Costanzo et al., 2016; Milton et al., 2004; Patel et al., 2013). These differences may reflect training-related plasticity, pre-existing traits that facilitate progression to elite sport, or combinations of these factors. Applying general-population reference values to elite athletes could therefore misclassify population-specific variation as clinically significant.

Additionally, repeated concussions and sub concussive impacts in contact sports may be associated with structural or microstructural brain changes, and findings across athlete imaging studies have been heterogeneous (Churchill et al., 2017a,b; Koerte et al., 2015; Mills et al., 2020; Zivadinov et al., 2018). The influences of elite performance, concussion history, and cumulative head-impact exposure may overlap and act in opposing directions. Normative modeling offers a way to estimate an individual's position relative to an expected distribution while accounting for relevant covariates, but its validity depends on the composition, size, and transportability of the reference sample (Bozek et al., 2023; Marquand et al., 2016; Rutherford et al., 2023). Few studies have tested whether elite athletes systematically deviate from general-population structural references or whether those deviations relate to performance, sensorimotor function, or reported concussion history.

One common measure of brain morphology is regional brain volume. Atlas-based segmentation has historically been used to estimate regional volumes by aligning a labeled template to an individual's scan (Avants et al., 2011). More recent automated approaches use machine-learning methods to reduce processing time and improve scalability. BrainKey (http://brainkey.ai) provides regional brain volumes expressed relative to age- and sex-referenced distributions. In the present study, these externally generated percentile measures were used to examine coordinated multiregional variation. This approach does not establish or validate an athlete-specific normative model. Instead, it uses population-referenced regional percentiles as a common measurement scale to examine whether coordinated structural profiles in elite athletes are associated with performance, sensorimotor function, and reported concussion history.

In this exploratory study, we used age- and sex-referenced regional brain-volume percentiles to characterize multiregional structural variation in elite athletes. Our objective was to evaluate whether sample-derived structural profiles were associated with expert-rated athletic performance, objective sensorimotor phenotypes derived from KINARM robotic assessment, and reported concussion history. The study was not designed to determine whether athletes differ structurally from non-athletes, establish an athlete-specific baseline of brain health, or validate an athlete-specific normative model. Rather, it sought to identify candidate associations that could inform future longitudinal studies and studies incorporating appropriately matched non-athlete comparison groups.

## 2 Materials and methods

### 2.1 Participants

Fifty-one active and retired elite athletes were recruited between June 15, 2016 and September 27, 2017. All athletes were patients of one of the authors (ML) and were recruited through text, telephone, or email contact describing the study and gauging interest. This direct-contact recruitment approach and the associated consent procedures were explicitly reviewed and approved by the Institutional Review Boards of the Medical University of South Carolina and Queen's University. Potential participants were informed that participation was voluntary, that declining or withdrawing would not affect their clinical care or relationship with their treating clinicians, and that no clinical services or benefits depended on participation. Clinical care decisions were kept separate from decisions regarding research participation. Written informed consent was obtained before any study procedures by ML after participants had an opportunity to review the study information and ask questions.

Participants were eligible if they had competed at the professional or international elite level for at least 2 years. Exclusion criteria included metabolic disease (e.g., diabetes), cardiovascular disease, severe respiratory disease, current smoking, another serious medical condition that could increase study risk, and standard MRI contraindications. All participants completed a medical history review and standardized MRI safety screening. Each participant underwent neuroimaging, kinesiological, neuropsychological testing, and evaluation for history of concussion. No participants were excluded because of missing imaging data. Cases with incomplete behavioral data were excluded listwise from analyses involving those variables. All study procedures were reviewed and approved by the IRB of the Medical University of South Carolina and Queen's University. Study activities were conducted in accordance with the Declaration of Helsinki.

### 2.2 Imaging

Neuroimaging was conducted on a 3T Siemens Prisma Fit. The protocol consisted of T1-weighted (T1), Diffusion Weighted (DWI), Susceptibility Weighted (SWI), and functional MRI (fMRI). For the purposes of the current study, only the T1 was used, obtained using a Magnetization-Prepared Rapid Gradient Echo (MPRAGE) sequence with the following parameters: TR/TE = 2250/4ms, TI = 925ms, Flip Angle = 9, Matrix = 256 x 256, FOV = 25.6cm, Slice Thickness = 1mm, Resolution = 1 x 1 x 1mm).

### 2.3 Brain segmentation and normative percentile estimation

Structural MRI data were uploaded to BrainKey for automated segmentation into 25 predefined measures: total and left/right hippocampus; total and left/right lateral ventricles; total and left/right inferior lateral ventricles; hippocampal occupancy; third ventricle; fourth ventricle; total ventricles; frontal, parietal, occipital, and temporal gray matter; caudate; putamen; amygdala; thalamus; white matter; brainstem; globus pallidus; and cerebellum. BrainKey's KeyLayer segmentation algorithm was trained on 800 manually segmented ground-truth datasets and evaluated on an independent set of 100 manually labeled datasets (overall Dice coefficient = 0.92) (Williamson et al., 2025). Region-specific validation estimates were not available for the software version used in this study.

Regional volumes were normalized to total segmented brain volume to reduce the influence of overall head size. Age- and sex-referenced percentiles were generated from an external reference resource comprising 3,564 participants aged 6–93 years, with an approximate sex distribution of 53% male and 47% female. The reference data included T1-weighted scans acquired across Siemens, GE, and Philips systems at 1.5T and 3T and across multiple imaging sites. Scanner manufacturer, field strength, acquisition site, and sequence parameters were not included as explicit covariates in the percentile calculation. The diversity of the reference data broadens the range of represented imaging conditions but does not establish transportability to the present athlete cohort. All athlete scans were acquired using the same single-site 3T MPRAGE protocol and processed through BrainKey's automated image-quality and segmentation workflow. All 51 scans produced usable segmentation and percentile outputs. No scans were excluded from the imaging analyses or manually corrected.

To reduce redundancy, total bilateral measures were removed, leaving 18 regions for statistical analysis: left and right hippocampus; left and right lateral ventricle; third and fourth ventricles; frontal, parietal, temporal, and occipital gray matter; caudate; putamen; amygdala; thalamus; white matter; brainstem; globus pallidus; and cerebellum. Each participant's normalized regional volume was expressed as an age- and sex-referenced percentile relative to the external reference population. These percentile scores served as the primary brain-derived measures.

### 2.4 Principal component analysis of normative regional percentiles

The 18 age- and sex-referenced regional percentile scores generated by Brain Key were entered into PCA to identify dominant multivariate patterns of coordinated structural variation rather than examining each region independently. Before PCA, all regional percentile measures were mean-centered and scaled to unit variance. Components were retained based on eigenvalues greater than 1 together with inspection of the scree plot (Supplementary Figure 1). Loadings shown in Table 1 have an absolute magnitude ≥0.20. The resulting PCA scores were used as the primary neuroimaging predictors in subsequent statistical analyses.

**Table 1:** Brain-region loadings for the five retained principal components (*N* = 51). Only |loading| ≥0.20 are shown; blanks indicate loadings below the display threshold.

PC1 (22.3%)	PC2 (21.6%)	PC3 (10.6%)	PC4 (9.6%)	PC5 (7.3%)
Brainstem (0.403)	Right lateral ventricle (0.373)	White matter (0.634)	Putamen (0.403)	Amygdala (-0.486)
Right hippocampus (0.369)	Temporal gray matter (−0.372)	Parietal gray matter (−0.400)	Fourth ventricle (0.396)	Fourth ventricle (−0.387)
Globus pallidus (0.363)	Left lateral ventricle (0.354)	Occipital gray matter (−0.331)	Globus pallidus (0.338)	Thalamus (0.378)
Left hippocampus (0.349)	Third ventricle (0.337)	Cerebellum (−0.284)	Right hippocampus (−0.303)	Caudate (−0.342)
Cerebellum (0.304)	Parietal gray matter (−0.316)	Thalamus (−0.253)	Cerebellum (−0.293)	Right lateral ventricle (−0.329)
Putamen (0.298)	Frontal gray matter (−0.291)		Occipital gray matter (0.270)	Temporal gray matter (−0.312)
Occipital gray matter (−0.273)	Amygdala (−0.228)		Left hippocampus (−0.246)	Left lateral ventricle (−0.251)
Left lateral ventricle (−0.233)	Thalamus (0.213)		Frontal gray matter (−0.237)	Occipital gray matter (0.213)
			Caudate (−0.232)	
			White matter (0.222)	

### 2.5 Bootstrap assessment of PCA stability

To evaluate the stability of the sample-derived brain components, we performed a non-parametric bootstrap analysis with 2,000 resamples. In each bootstrap iteration, participants were sampled with replacement, and PCA was repeated using the same 18 age- and sex-referenced regional percentile measures, with variables centered and scaled within each resample. Stability was summarized using the median congruence between original and bootstrap loading vectors, percentile-based 95% bootstrap intervals for each regional loading, and the proportion of bootstrap samples in which each loading retained the same sign as the original solution. Components with higher congruence and consistently signed loadings were interpreted as more stable. Congruence coefficients were interpreted according to previous guidance, where values 0.85–0.94 suggest fair similarity and values >0.95 indicate high similarity or practical equivalence between loading patterns (Lorenzo-Seva and Ten Berge, 2006). Components with higher congruence and consistent sign loadings were interpreted as more internally stable, whereas components with lower congruence were interpreted more cautiously.

### 2.6 Performance rating model

Participants were rated on a study-specific performance scale from 1 to 5 (1 = highest performance, 5 = lowest performance) by an expert in sports performance and sports medicine (ML). ML had a pre-existing clinical relationship with the participants because they had received care through his practice. Ratings were based on knowledge of each athlete's competitive level and accomplishments and were assigned without access to the brain volumetric or KINARM results. The scale was developed for this study, was not independently validated, and was assigned by a single rater, so inter-rater reliability could not be assessed. The categories were: 1 = athletes considered to be in the top 5% of their sport, including Olympic medalists or international champions; 2 = consistent international competitors with strong results; 3 = national champions or consistent contenders; 4 = state/provincial-level athletes with potential to achieve higher levels; and 5 = athletes who met the study eligibility criteria but whose competitive performance was judged comparable to a high-level collegiate standard. The performance categories described relative competitive achievement and did not define study eligibility. All participants, including those assigned rating 5, met the requirement of at least 2 years of professional- or international-level competition. The original counts were 9, 3, 15, 3, and 21 for ratings 1 through 5, respectively. To improve category size, the scale was collapsed into three ordered groups: high performance (ratings 1-2; n = 12), intermediate performance (rating 3; *n* = 15), and lower performance (ratings 4–5; *n* = 24). The ordered outcome was coded Low < Mid < High.

The relationship between brain structural components and the ordered three-level performance outcome was assessed with a Bayesian cumulative-logit model. Brain PC1-PC5 and sex were entered simultaneously as predictors. The model used Normal (0, 0.5) priors for slopes and Normal (0, 2) priors for thresholds/intercepts. Four Markov chains were run for 4,000 iterations each, including 2,000 warmup iterations, with adapt_delta = 0.97 and seed = 123. Posterior means, 95% credible intervals, and posterior probability of direction (Pr (direction)) were used to summarize uncertainty. Sensitivity analyses were conducted to evaluate whether the association between brain structure and performance depended on the definition of the study-specific performance groups. Alternative outcomes included the original 5-point expert rating, a binary high-performance classification comparing ratings 1–2 with 3–5, and a top-performance classification comparing rating 1 with ratings 2–5. All sensitivity models used the same Bayesian framework as the primary performance model, with Brain PC1–PC5 and sex entered as predictors.

Because hockey represented the largest individual sport category and was unevenly distributed across performance groups, two *post hoc* sport-composition sensitivity analyses were conducted. Sport information was available for 49 participants. First, the primary cumulative-logit performance model was repeated with hockey vs. non-hockey status included as an additional covariate alongside Brain PC1–PC5 and sex. Second, the primary model was repeated after excluding hockey athletes. Both models used the same priors, sampling parameters, and outcome definition as the primary performance model. More detailed adjustment by individual sport was not performed because the non-hockey participants were distributed across numerous sparsely represented sports. Contact/collision classification, active-vs.-retired status, years of elite participation, and quantitative head-impact exposure were not available in a sufficiently complete and standardized form for additional modeling.

### 2.7 KINARM sensorimotor models

KINARM robotic assessment was completed by 44 participants to characterize upper-limb sensorimotor performance. Participants completed a battery of standard KINARM tasks, including arm-position matching, ball-on-bar, object hit, object hit-and-avoid, and visually guided reaching tasks. Extracted outcomes included task- and movement-level summary scores, total and side-specific object hits, distractor errors, processing rate, hand speed, posture speed, reaction time, movement time, path-length ratio, task completion time, dwell time, and error counts. Where applicable, outcomes were retained separately for the left and right upper limbs. A total of 56 KINARM-derived variables were included, and participants with incomplete KINARM data were excluded from the sensorimotor PCA. Variables were mean-centered and scaled to unit variance before PCA. The first nine components explained 72% of the total variance. Subsequent analyses were restricted to the first five KINARM components based on the elbow in the scree plot (Supplementary Figure 2). To assess the internal stability of the KINARM PCA solution, we performed a non-parametric bootstrap analysis with 2,000 resamples. Participants were sampled with replacement, and PCA was repeated using the same 56 KINARM variables, with variables centered and scaled within each resample. Stability was summarized using the median and percentile-based 95% bootstrap interval of the loading-vector congruence, the distribution of variance explained by each matched component, percentile-based intervals for individual loadings, and the proportion of bootstrap samples in which each loading retained the direction of the original loading. These analyses assess internal resampling stability and do not establish external reproducibility. Separate Bayesian Gaussian regression models were fit for the analyzed KINARM PCs, with Brain PC1-PC5 and sex entered simultaneously. Slope priors were Normal (0, 0.5) and the intercept prior was Normal (0, 1.5). Models used four chains, 4,000 iterations, 2,000 warmup iterations, adapt_delta = 0.99, maximum tree depth = 15, and seed = 123.

### 2.8 Concussion history model

Concussion history was defined as a reported history of concussion diagnosed by a practicing medical professional according to contemporary consensus criteria for sport-related concussion (McCrory et al., 2013). No participants reported loss of consciousness associated with concussive injuries. This classification yielded 23 participants with a reported history of concussion and 27 without a reported history of concussion (*N* = 50; 1 participant with missing data). A Bayesian Bernoulli-logit model was fit with concussion history as the binary outcome and Brain PC1-PC5 plus sex as simultaneous predictors. Slope priors were Normal (0, 0.5) and the intercept prior was Normal (0, 1.5). The model used four chains, 4,000 iterations, 2,000 warmup iterations, adapt_delta = 0.99, maximum tree depth = 15, and seed = 123. Posterior predictive checks were used to assess model fit. All analyses were conducted in R 4.4.0 using brms (2.22.0) for Bayesian modeling.

### 2.9 Prior specification

Continuous predictors were standardized before Bayesian modeling, so slope coefficients represent the expected change in the outcome per 1-SD increase in the predictor. Normal (0, 0.5) priors were used for slope parameters as weakly regularizing priors, centering effects near zero while still allowing small-to-moderate associations. For logistic and cumulative-logit models, this prior corresponds approximately to a prior expectation that a 1-SD predictor change would usually be associated with odds ratios between 0.38 and 2.66, while still permitting larger effects if supported by the data. Threshold/intercept priors were specified more broadly, using Normal (0, 2) priors for ordinal thresholds and Normal (0, 1.5) priors for Gaussian and Bernoulli-logit intercepts, to allow flexible baseline outcome distributions while maintaining computational stability in a modest sample.

### 2.10 Interpretation of bayesian evidence and multiplicity

Before inspection of the model results, a posterior probability of direction of at least 0.95 was prespecified as a descriptive threshold for identifying coefficients for focused discussion. Pr (direction) represents the proportion of the posterior distribution having the same sign as the posterior point estimate. Thus, a value of 0.95 indicates that 95% of the posterior mass supports one coefficient direction. This threshold was used to organize the presentation of results rather than to define statistical significance. Because multiple brain components were evaluated across performance, KINARM, and concussion-history models, Pr (direction) was not interpreted as a multiplicity-adjusted probability or as confirmatory evidence. To reduce selective reporting, all modeled coefficients, 95% credible intervals, and Pr (direction) values are reported in the Supplementary Material, regardless of whether they met the pre specified descriptive threshold. Interpretation considered the magnitude and uncertainty of each estimate, whether the 95% credible interval included the null, component stability, and consistency across sensitivity analyses rather than Pr (direction) alone. Given the exploratory design, modest sample size, heterogeneous outcome types, and absence of pre specified cross-outcome exchangeability assumptions, a single hierarchical model pooling coefficients across the performance, KINARM, and concussion analyses was not feasible.

## 3 Results

### 3.1 Participant characteristics

Performance group counts were High (*n* = 12), Intermediate (*n* = 15), and Low (*n* = 24). The sample included 43 men (84%) and 8 women (16%). Sex was included as a covariate in the Bayesian models. Age did not differ significantly among the three performance groups (Kruskal–Wallis χ^2^ (2) = 2.13, *p* = 0.345). Sport information was available for 49 participants. Hockey was the largest sport category (*n* = 22, 45%) and was unevenly distributed across performance groups, representing 42% of the high-performance group, 80% of the intermediate-performance group, and 23% of the lower-performance group. Participant characteristics are shown in Table 2.

**Table 2:** Demographic and concussion characteristics by performance group.

Characteristic	Overall (*N* = 51)	High (*N* = 12)	Mid (*N* = 15)	Low (*N* = 24)	*p*-value
**Age, years**	35.2 ± 14.9	32.5 ± 11.1	40.4 ± 16.3	33.3 ± 15.4	0.345
**Sex**					0.216
F	8 (16%)	4 (33%)	1 (7%)	3 (12%)	
M	43 (84%)	8 (67%)	14 (93%)	21 (88%)	
**Sport (*****N*** **=** **49)**					0.052
Baseball	1 (2%)	0 (0%)	0 (0%)	1 (5%)	
Basketball	3 (6%)	0 (0%)	0 (0%)	3 (14%)	
Bobsled	1 (2%)	1 (8%)	0 (0%)	0 (0%)	
Football	5 (10%)	0 (0%)	2 (13%)	3 (14%)	
Hockey	22 (45%)	5 (42%)	12 (80%)	5 (23%)	
Lacrosse	2 (4%)	0 (0%)	0 (0%)	2 (9%)	
MMA	1 (2%)	0 (0%)	0 (0%)	1 (5%)	
Rugby	3 (6%)	1 (8%)	0 (0%)	2 (9%)	
Skiing/skating/snow	5 (10%)	2 (17%)	0 (0%)	3 (14%)	
Soccer	1 (2%)	0 (0%)	0 (0%)	1 (5%)	
Swimming	2 (4%)	1 (8%)	0 (0%)	1 (5%)	
Track/running	2 (4%)	1 (8%)	1 (7%)	0 (0%)	
Trampoline	1 (2%)	1 (8%)	0 (0%)	0 (0%)	
**Concussion history (*****N*** **=** **50)**					0.478
No concussion history	27 (54%)	8 (67%)	6 (43%)	13 (54%)	
Concussion history	23 (46%)	4 (33%)	8 (57%)	11 (46%)	

Data are mean ± SD or *n* (%). *p*-values compare Low, Mid, and High performance groups. Age was tested using the Kruskal-Wallis rank-sum test. Categorical variables were tested using Pearson chi-square tests when expected cell counts were adequate, or Monte Carlo simulated chi-square tests when expected cell counts were sparse. ^*^ Indicates *p* < 0.05.

### 3.2 Brain region components

PCA of the 18 age- and sex-referenced regional percentile measures yielded five retained components that together explained 71.4% of total variance (PC1 = 22.3%, PC2 = 21.6%, PC3 = 10.6%, PC4 = 9.6%, and PC5 = 7.3%). The corresponding eigenvalues were 4.02, 3.88, 1.91, 1.73, and 1.31. Regional loadings with absolute magnitude ≥0.20 are shown in Table 1.

### 3.3 Bootstrap stability of the brain components

Across 2,000 bootstrap resamples, median loading-vector congruence with the original solution was 0.855 for PC1, 0.869 for PC2, 0.749 for PC3, 0.758 for PC4, and 0.664 for PC5. Using established guidance for Tucker congruence coefficients, PC1 and PC2 showed fair loading-pattern similarity under resampling, whereas PC3–PC5 showed weaker stability. Overall, the bootstrap results support stronger internal reproducibility for PC1–PC2 and more cautious interpretation of PC3–PC5. Full component- and loading-level stability estimates are provided in Supplementary Tables 1A, and 1B.

### 3.4 Brain structure and athletic performance

**Figure 1:**
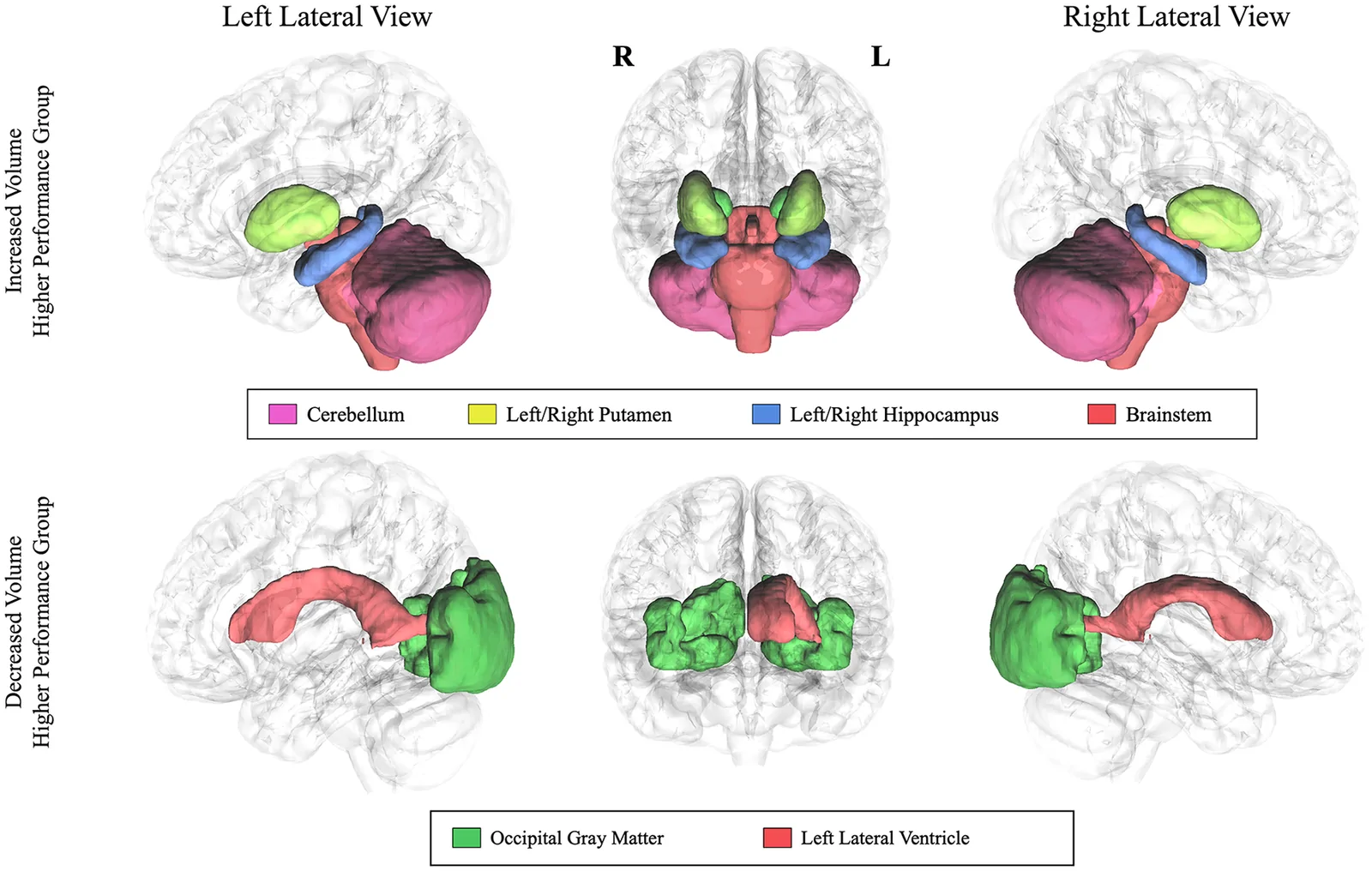
Brain PC1 loading pattern related to athletic performance. Regions corresponding to higher age- and sex-referenced volume percentiles in the higher-performance group (top row), and regions corresponding to lower age- and sex-referenced volume percentiles in the higher-performance group (bottom row). Top-row colors denote cerebellum (pink), left/right putamen (yellow), left/right hippocampus (blue), and brainstem (red). Bottom-row colors denote occipital gray matter (green) and left lateral ventricle (red). PC, principal component.

**Figure 2:**
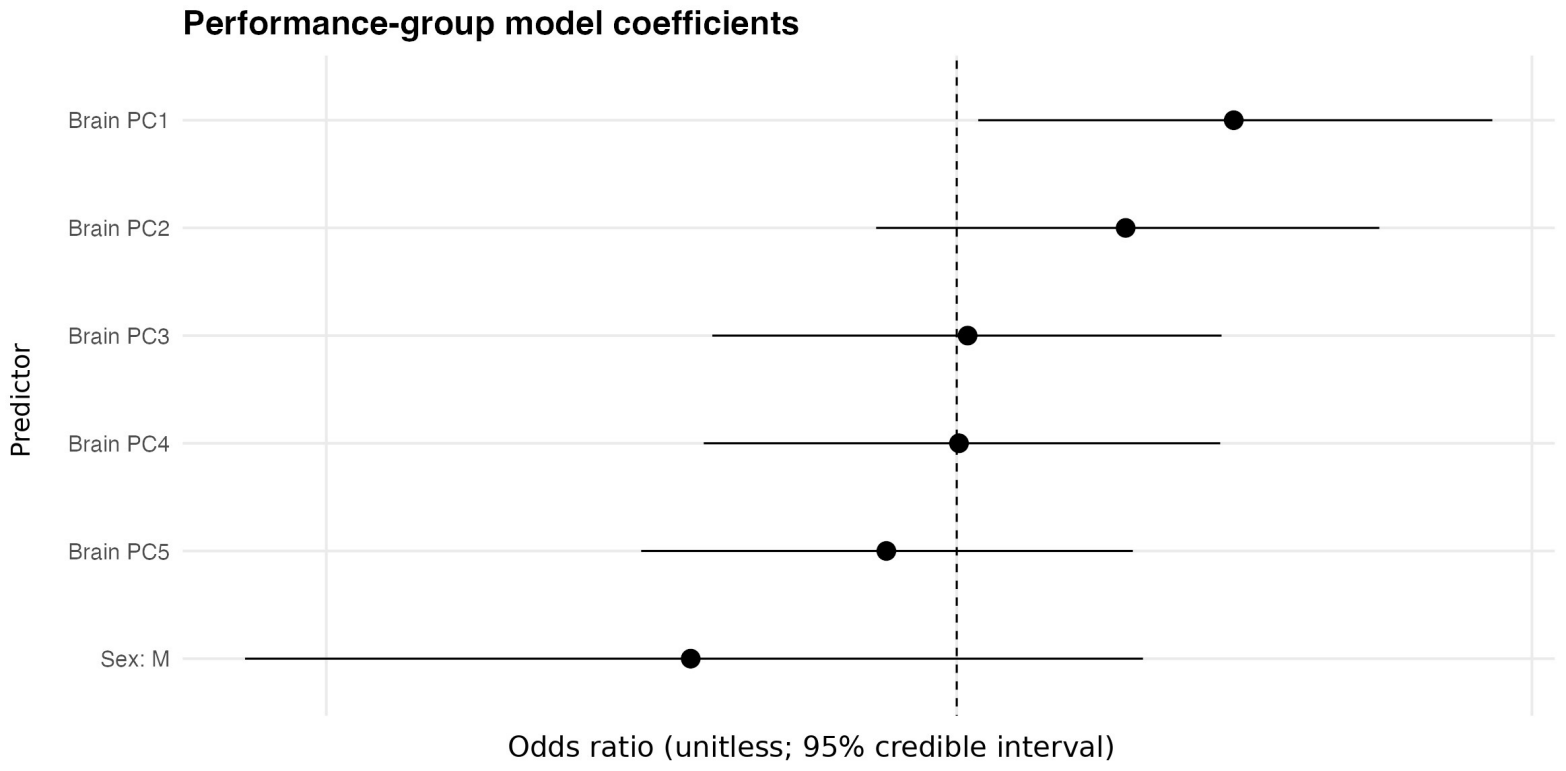
Bayesian model coefficients for the association between Brain principal components (PCs) and athletic performance group. Forest plot showing odds ratios and 95% credible intervals from the Bayesian cumulative-logit model evaluating the association between Brain PC1–PC5 and ordered performance group, adjusted for sex (*N* = 51). The dashed vertical line indicates an odds ratio of 1. Odds ratios greater than 1 indicate higher odds of being in a higher performance group.

In the cumulative-logit model including Brain PC1-PC5 and sex, Brain PC1 had a positive coefficient for higher performance-group membership [β = 0.53, 95% CrI [0.04, 1.02], Pr (direction) = 0.983, *n* = 51; OR = 1.70, 95% CrI (1.04, 2.78)]. No other coefficient in the primary performance model met the prespecified Pr (direction) threshold. Higher Brain PC1 scores reflected higher age- and sex-referenced percentiles in brainstem, bilateral hippocampi, globus pallidus, cerebellum, and putamen, together with lower percentiles in occipital gray matter and the left lateral ventricle (Figure 1). All model coefficients are presented in Supplementary Table 3 and Figure 2.

Sensitivity analyses suggested that the Brain PC1 association was not entirely dependent on the primary three-level performance grouping. The association persisted when the original five-level expert rating was modeled as an ordered outcome (OR = 1.69, 95% CrI: 1.05–2.73, Pr (direction) = 0.98) and in the high-performance binary model comparing ratings 1–2 with ratings 3–5 (OR = 1.68, 95% CrI: 0.94–3.06, Pr (direction) = 0.96). The association was weaker when performance was defined only by the top rating (rating 1 vs. 2–5; OR = 1.33, 95% CrI: 0.72–2.50, Pr (direction) = 0.82). Full sensitivity results are provided in Supplementary Table 4A.

Sport-composition sensitivity analyses indicated that the Brain PC1 estimate was attenuated after accounting for hockey participation. In the model adjusted for hockey vs. non-hockey, the Brain PC1 coefficient remained positive but its credible interval included the null [β = 0.49, 95% CrI (−0.03, 1.02), OR = 1.63, 95% CrI (0.97, 2.76), Pr (direction) = 0.97, *n* = 49]. The hockey coefficient itself was uncertain [OR = 1.39, 95% CrI (0.62, 3.04), Pr (direction) = 0.79]. When the model was restricted to the 27 non-hockey athletes, the Brain PC1 estimate remained directionally positive but was smaller and more uncertain [β = 0.31, 95% CrI (−0.33, 0.96), OR = 1.36, 95% CrI (0.72, 2.60), Pr (direction) = 0.82]. These analyses suggest that the primary performance result was not wholly attributable to the inclusion of hockey athletes, but they do not establish an association independent of sport composition. Complete results are provided in Supplementary Table 4B.

### 3.5 Brain structure and KINARM sensorimotor performance

**Figure 3:**
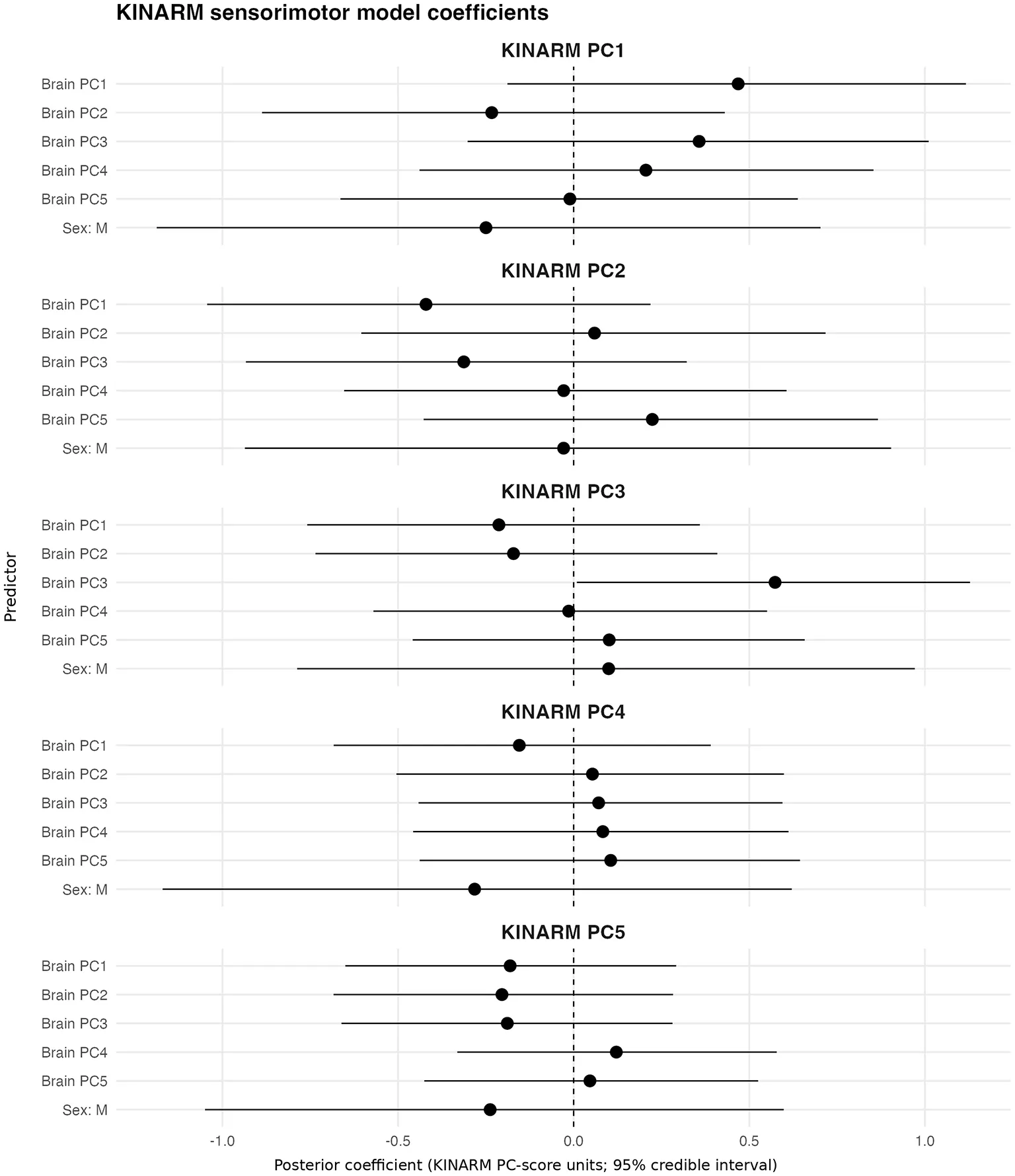
Bayesian model coefficients for the association between Brain principal components (PCs) and KINARM sensorimotor PCs. Forest plots showing posterior mean coefficients and 95% credible intervals from Bayesian Gaussian models evaluating the association between Brain PC1–PC5 and KINARM PC1–PC5, adjusted for sex (*N* = 44). Each panel represents a separate KINARM PC outcome. The dashed vertical line indicates a coefficient of 0. Coefficients greater than 0 indicate higher values of the corresponding KINARM PC.

The first nine KINARM components explained 72.1% of the total variance, and the first five components, which were retained for subsequent modeling based on the scree-plot elbow, explained 55.0%. Bootstrap analysis demonstrated limited stability of the KINARM component solution. Median loading-vector congruence was 0.819 for KINARM PC1, 0.771 for PC2, 0.721 for PC3, 0.698 for PC4, and 0.492 for PC5. In the Gaussian regression model including Brain PC1–PC5 and sex, Brain PC3 met the prespecified descriptive Pr(direction) threshold for KINARM PC3 [β = 0.57, 95% CrI [0.01, 1.13], Pr (direction) = 0.978, *n* = 44]. However, both Brain PC3 and KINARM PC3 showed limited bootstrap stability. Several of the largest KINARM PC3 loadings nevertheless retained their original direction in most bootstrap samples, including the Object Hit-and-Avoid standardized score, task score, composite task score, and left-hand speed. Thus, individual loadings showed directional consistency, but the component-level solution was not sufficiently stable to support a robust interpretation of KINARM PC3 as a reproducible sensorimotor phenotype. This result is therefore considered highly exploratory and potentially dependent on the sample-specific component solutions. Higher Brain PC3 scores reflected higher age- and sex-referenced percentiles in white matter together with lower percentiles in parietal gray matter, occipital gray matter, cerebellum, and thalamus. The remaining KINARM models had no coefficients with Pr (direction) ≥ 0.95. Complete model coefficients are presented in Supplementary Table 5 and Figure 3. Complete component- and loading-level stability results are provided in Supplementary Tables 2A, and 2B, and the original KINARM loadings are provided in Supplementary Table 2C.

### 3.6 Brain structure and concussion history

**Figure 4:**
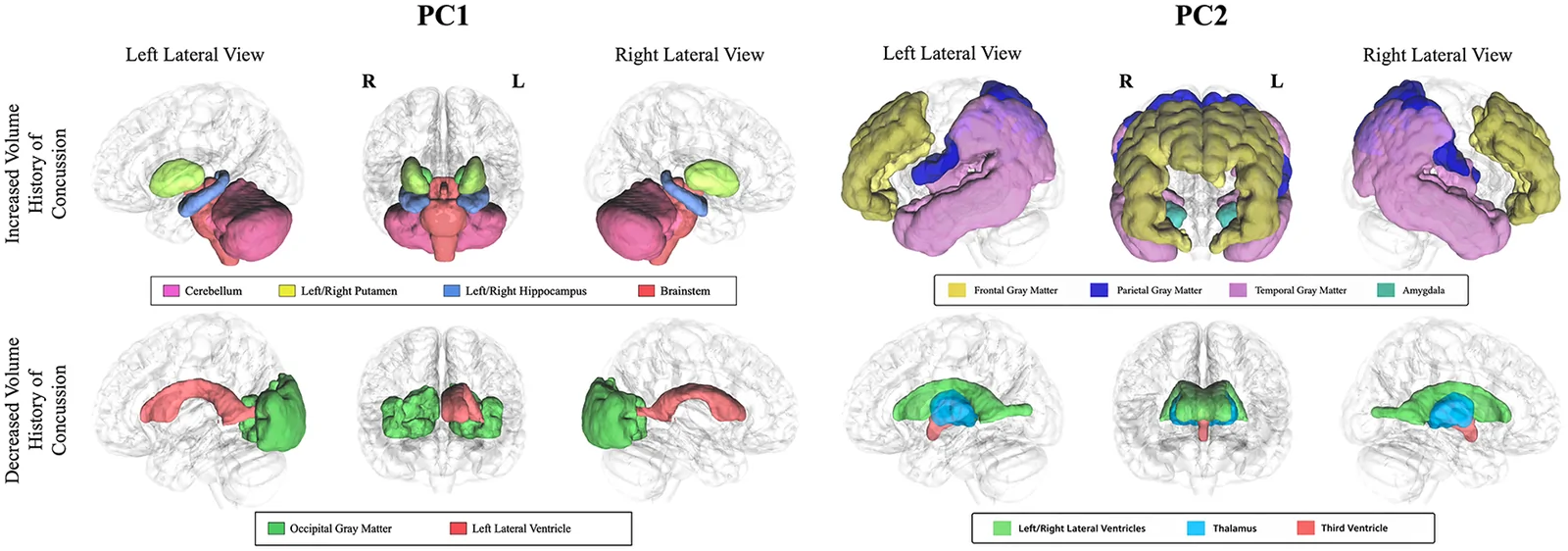
Brain PC1 and PC2 loading patterns related to reported concussion history. Regions corresponding to higher age- and sex-referenced volume percentiles in participants with reported concussion history are shown in the top row of each panel, and regions corresponding to lower age- and sex-referenced volume percentiles in participants with reported concussion history are shown in the bottom row of each panel. **Left Panel:** Brain PC1 regions associated with reported concussion history. Top-row colors denote cerebellum (pink), left/right putamen (yellow), left/right hippocampus (blue), and brainstem (red). Bottom-row colors denote occipital gray matter (green) and left lateral ventricle (red). **Right Panel:** Brain PC2 regions associated with reported concussion history. Because reported concussion history was associated with lower Brain PC2, the displayed regions reflect the inverse of the PC2 loading pattern. Top-row colors denote frontal gray matter (yellow), parietal gray matter (blue), temporal gray matter (pink), and amygdala (teal). Bottom-row colors denote left/right lateral ventricles (green), thalamus (blue), and third ventricle (red). PC, principal component.

**Figure 5:**
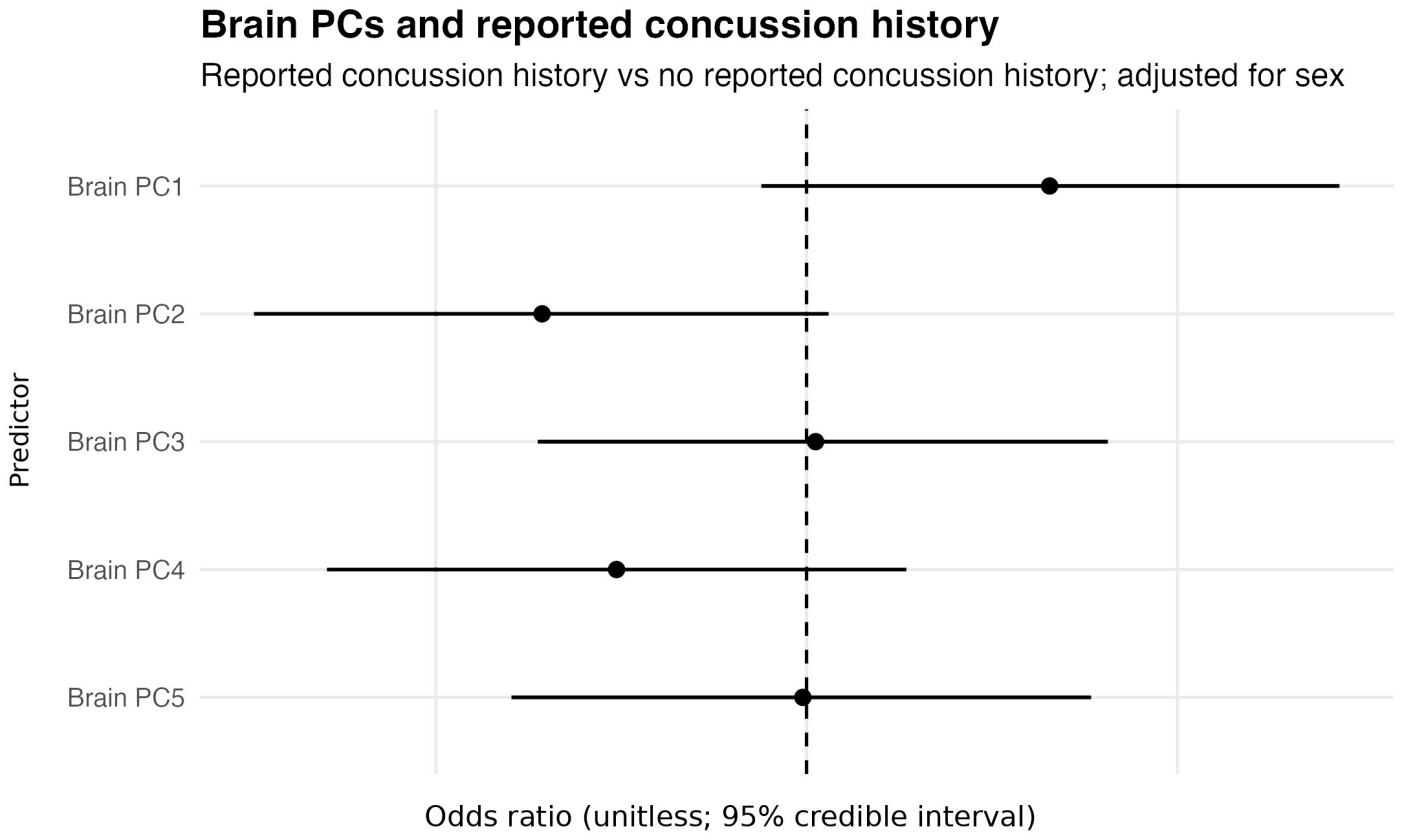
Bayesian model coefficients for the association between Brain principal components (PCs) and reported concussion history. Forest plot showing odds ratios and 95% credible intervals from the Bayesian Bernoulli-logit model evaluating the association between Brain PC1–PC5 and reported concussion history, adjusted for sex (*N* = 50). The dashed vertical line indicates an odds ratio of 1. Odds ratios greater than 1 indicate higher odds of reported concussion history.

Reported concussion history showed suggestive directional evidence for lower Brain PC2 [OR = 0.61, 95% CrI (0.36, 1.04), Pr (direction) = 0.97] and higher Brain PC1 (OR = 1.57, 95% CrI [0.92, 2.71], Pr (direction) = 0.95). Brain PC2 was characterized by higher age- and sex-referenced percentiles in the bilateral lateral ventricles, third ventricle, and thalamus, together with lower percentiles in temporal, parietal, and frontal gray matter and the amygdala. The negative coefficient indicates that concussion history was directionally related to the opposite component pattern: lower ventricular and thalamic percentiles and higher temporal, parietal, and frontal gray-matter and amygdala percentiles (Figure 4, right panel). The positive Brain PC1 coefficient indicates that concussion history was directionally related to higher percentiles in the brainstem, bilateral hippocampi, globus pallidus, cerebellum, and putamen, together with lower percentiles in occipital gray matter and the left lateral ventricle (Figure 4, left panel). Brain PC4 showed a weaker directionally negative association (OR = 0.70, 95% CrI: 0.41–1.20, Pr (direction) = 0.90), whereas Brain PC3 and Brain PC5 showed little evidence of association. Full model coefficients are provided in Supplementary Table 6, and Figure 5.

### 3.7 Model diagnostics

Across the reported Bayesian models, the maximum R-hat was 1.002, the minimum bulk effective sample size was 3,326, and the minimum tail effective sample size was 4,609, indicating satisfactory convergence and sampling efficiency. Posterior predictive checks did not identify major discrepancies between the fitted models and the observed outcome distributions. These diagnostics assess computation and model fit but do not address multiplicity or establish confirmatory evidence.

## 4 Discussion

This exploratory study used age- and sex-referenced regional brain-volume percentiles as a population-referenced measurement framework to examine multiregional structural variation in elite athletes. The analyses identified candidate relationships between sample-derived brain structural components and expert-rated athletic performance, KINARM-derived sensorimotor performance, and reported concussion history. Brain PC1 showed the clearest relationship with ordered performance group. Brain PC3 showed a positive relationship with KINARM PC3, although both components had limited bootstrap stability and this result should be considered highly exploratory. Reported concussion history showed suggestive directional relationships with lower Brain PC2 and higher Brain PC1. These findings do not demonstrate athlete-specific structural adaptation, injury-related change, or clinically meaningful abnormality. They instead identify multiregional patterns that require external evaluation in larger, longitudinal cohorts with objective exposure measures and matched non-athlete comparisons.

### 4.1 Brain phenotypes associated with athletic performance

The clearest performance-related signal was the positive association between Brain PC1 and ordered performance group. PC1 represented coordinated variation characterized by higher age- and sex-referenced percentiles in brainstem, bilateral hippocampi, globus pallidus, cerebellum, and putamen, together with lower percentiles in occipital gray matter and the left lateral ventricle. The distribution of positive loadings is broadly consistent with systems implicated in motor sequencing, timing, action selection, error correction, and spatial-contextual processing (Doyon et al., 2009; Penhune and Steele, 2012; Shadmehr and Krakauer, 2008). These functional interpretations are indirect, however, because PCA loadings reflect covariance among regional measures rather than independently tested regional effects.

The bootstrap findings provide some support for the internal stability of PC1. Its median loading-vector congruence was 0.855, and the major positive loadings showed high same-sign replication frequencies. Nevertheless, the bootstrap intervals for the occipital gray-matter and left lateral-ventricle loadings crossed zero, so these individual contributions should be interpreted cautiously. More generally, regional volume cannot be assumed to indicate neural efficiency, specialization, or pathology. The observed pattern could reflect training-related plasticity, pre-existing traits, selection into elite sport, sport composition, or sampling variability.

Sport composition is an important alternative explanation for the performance-related finding. Hockey constituted nearly half of the participants with known sport and was particularly common in the intermediate-performance group. After adjustment for hockey status, the Brain PC1 coefficient remained directionally positive but was attenuated. The estimate was smaller and more uncertain when the analysis was restricted to non-hockey athletes. These results do not indicate that hockey participation fully explains the primary association, but they also do not demonstrate that the association is independent of sport. Brain PC1 may capture some combination of performance level, sport-specific selection, training demands, cumulative participation, head-impact opportunity, or other characteristics that differ among sports.

### 4.2 Relationship to sensorimotor measures

Within the original sample, KINARM PC3 was characterized primarily by Object Hit and Object Hit-and-Avoid measures, including positive loadings for standardized, task, and composite scores and negative loadings for bilateral hand speed. This pattern may reflect variation in rapid visuomotor decision-making, inhibitory control, or speed–accuracy strategy. Such processes depend on distributed neural systems involved in skilled motor control, action selection, error correction, and visuomotor adaptation (Doyon et al., 2009; Penhune and Steele, 2012; Shadmehr and Krakauer, 2008). However, these functional interpretations are indirect and should be considered tentative because the KINARM component was derived from many variables relative to the available sample.

Bootstrap analysis confirmed limited stability of KINARM PC3. Its median loading-vector congruence was 0.721, with a broad 95% bootstrap interval of 0.267–0.939. Although several of the largest Object Hit and Object Hit-and-Avoid loadings retained their directions in most resamples, the overall component pattern was sensitive to participant resampling. Brain PC3, which showed a positive relationship with KINARM PC3 in the regression model, also demonstrated weaker bootstrap stability than Brain PC1 and PC2. The observed Brain PC3–KINARM PC3 relationship therefore depends on two sample-derived components with limited internal stability and should be considered highly exploratory.

The KINARM analysis may nevertheless provide a preliminary distinction between laboratory-based sensorimotor behavior and the broader expert-rated performance outcome. Brain PC1 was related to the holistic performance grouping, whereas Brain PC3 was related to KINARM PC3. The expert rating incorporated competitive achievement, consistency, and sport-specific performance, while the KINARM measures captured standardized upper-limb behavior under controlled conditions. These findings may therefore reflect partly different dimensions of athletic performance, but this interpretation is hypothesis-generating and requires testing in larger samples using externally validated component structures.

### 4.3 Reported concussion history and brain-volume profiles

Reported concussion history showed suggestive directional evidence for lower Brain PC2 and higher Brain PC1, suggesting that concussion history was related to multiregional structural profiles rather than a single isolated volumetric pattern. Prior athlete neuroimaging studies have reported heterogeneous structural findings across cohorts, sports, exposure definitions, and imaging methods (Bobholz et al., 2021; Churchill et al., 2017a,b; Echlin et al., 2021; Koerte et al., 2015, 2023; Major et al., 2021; Mills et al., 2020; Zivadinov et al., 2018). Consistent with this broader literature, the present findings should be viewed as hypothesis-generating: the available concussion measure captured reported history of medically diagnosed concussion but did not characterize injury timing, severity, recovery duration, or cumulative subconcussive exposure. Accordingly, these results identify candidate brain-volume profiles associated with concussion history in elite athletes, but do not support claims that individual regions were selectively protected, vulnerable, or degraded. The association of Brain PC1 with both higher performance-group membership and reported concussion history may reflect partial overlap among athletic expertise, cumulative competition exposure, sport type, and opportunity for head-impact events. Because detailed exposure measures were not available in this dataset, future studies should test whether this component reflects athletic performance, head-impact exposure, or shared variance between these factors. Sport-specific exposure may also confound the concussion-history findings. The cohort included athletes from sports with substantially different opportunities for collision, repetitive head impact, and concussion recognition. Detailed measures of playing position, years of exposure, cumulative head impacts, and timing of injuries were unavailable, preventing separation of concussion history from broader sport and exposure characteristics.

The present analyses were limited to macroscopic regional volumes derived from T1-weighted MRI. These measures do not directly assess axonal integrity, neuro inflammation, functional compensation, or other cellular and physiological processes that may be relevant to concussion and repetitive head-impact exposure. The observed profiles should therefore not be interpreted as evidence of a specific injury mechanism, neural resilience, or compensatory response.

### 4.4 Interpreting population-referenced brain profiles in elite athletes

The present findings highlight the interpretive complexity of applying general-population brain-volume references to elite athletes. This should not be interpreted as evidence of bias or error in the normative modeling approach itself. Rather, the observed association indicates that coordinated variation in population-referenced measures may be related to athletic performance, training history, sport composition, selection into elite sport, head-impact exposure, or sampling variability. The present cross-sectional data cannot distinguish among these possibilities or determine whether the observed variation is adaptive, adverse, or clinically meaningful. In this context, athlete-specific baselines may help distinguish stable population-specific structural profiles from clinically meaningful deviation or change. The present study did not construct or validate such a baseline, but the findings support future work using larger representative samples, matched non-athlete comparisons, sport- and sex-stratified reference data, external validation, and repeated within-athlete measurements.

More broadly, future studies could integrate structural MRI with diffusion and functional MRI, blood-based biomarkers, physiological monitoring, sensorimotor testing, objective head-impact measures, and longitudinal clinical assessments. Such multimodal and repeated-measures approaches may help distinguish stable athlete-associated variation from training-related change and injury-related vulnerability (Hellewell et al., 2021; Esopenko et al., 2023). The present study contributes an exploratory structural-imaging analysis, but any athlete-specific reference framework will require larger samples, matched comparisons, and external longitudinal validation.

### 4.5 Strengths and limitations

A notable strength of this study is the rarity and clinical relevance of the cohort: elite athletes spanning a range of performance levels and concussion histories, with multi-domain phenotyping that included structural imaging and robotic sensorimotor assessment. The study also moved beyond isolated regional comparisons by evaluating multiregional brain-structure profiles in relation to athletic performance, sensorimotor function, and reported concussion history. Methodologically, the use of age- and sex-referenced regional percentiles, PCA-based dimensionality reduction, Bayesian modeling, bootstrap assessment of PCA stability, and de identified case-level percentile displays provide a structured framework for exploratory analysis in a modest sample.

Several limitations warrant consideration. First, because Brain Key is proprietary, the complete software implementation, trained model parameters, and normative reference curves are not publicly available. Although we provide the available information regarding reference-sample composition, volume normalization, age- and sex-referencing, acquisition characteristics, quality control, and overall segmentation validation, region-specific accuracy and transportability of the population-referenced percentiles to elite athletes have not been independently established. These measures should therefore be interpreted as population-referenced indices rather than definitive indicators of structural normality, abnormality, adaptation, or injury. To improve interpretability, we provide de identified case-level percentile profiles and regional percentile-distribution plots, allowing readers to examine the participant-level patterns underlying the multivariate analyses. Second, the modest sample size relative to the number of regional measures increases the risk that some PCA components, particularly later components, may be sample-specific. Bootstrap analysis helped characterize internal stability, but it does not eliminate over fitting or replace external validation. Third, the cross-sectional design prevents determination of whether the observed profiles reflect pre-existing traits, training-related adaptation, concussion exposure, aging, retirement, or combinations of these factors. Longitudinal studies beginning before or early in elite sport participation, with repeated imaging, objective head-impact monitoring, detailed concussion timing and severity data, matched controls, and repeated performance and sensorimotor assessments, are needed to separate within-athlete change from stable between-athlete differences. Fourth, the performance scale was study-specific and assigned by a single expert rater who had a pre-existing clinical relationship with the participants. Although the rater was blinded to imaging and KINARM results, inter-rater reliability could not be assessed, and prior familiarity with participants may have influenced classification. Sensitivity analyses suggested that the Brain PC1 result was not entirely dependent on the three-level grouping, but they do not validate the underlying rating scale. Future studies should use prospectively defined criteria, multiple independent blinded raters, and objective sport-specific performance measures where feasible.

## 5 Conclusion

In this exploratory cohort of elite athletes, population-referenced multiregional brain-volume profiles showed candidate relationships with expert-rated athletic performance, KINARM-derived sensorimotor performance, and reported concussion history. These cross-sectional findings do not establish beneficial or pathological structural brain change and cannot distinguish effects of pre-existing traits, training, sport participation, head-impact exposure, concussion history, or sampling variability. The study neither constructs nor validates an athlete-specific normative model. Howecer, the identified profiles in this study provide a framework for external evaluation in larger, longitudinal cohorts incorporating matched non-athlete comparisons, objective exposure measures, and repeated within-athlete assessments.

## Data Availability

The datasets presented in this study can be found in online repositories. The names of the repository/repositories and accession number(s) can be found below: https://github.com/willi3by/elite_athlete_analysis.
