# Supplementary material for: Population-referenced multiregional brain-volume profiles associated with performance and concussion history in athletes: an exploratory study
Source: Front Hum Neurosci. 2026 Aug 10;20:1904810. doi: 10.3389/fnhum.2026.1904810 (PMC13500568; doi:10.3389/fnhum.2026.1904810)
Supplement: Supplementary file 1 [file Supplementary_File_1.docx]

Supplementary Material

**Supplementary Table S1A.** Component-level bootstrap stability of the brain principal-component solution. N = 51; 2,000 bootstrap resamples.

| **Component** | **Original variance (%)** | **Median congruence** | **IQR** | **95% bootstrap interval** | **Bootstrap variance, median (%)** | **Bootstrap variance, 95% interval (%)** |
| --- | --- | --- | --- | --- | --- | --- |
| PC1 | 22.35 | 0.855 | 0.753–0.926 | 0.633–0.979 | 23.50 | 17.07–30.41 |
| PC2 | 21.57 | 0.869 | 0.786–0.930 | 0.668–0.979 | 22.66 | 16.74–29.69 |
| PC3 | 10.60 | 0.749 | 0.640–0.840 | 0.321–0.940 | 11.06 | 6.71–14.82 |
| PC4 | 9.59 | 0.758 | 0.652–0.839 | 0.319–0.937 | 10.09 | 6.36–14.87 |
| PC5 | 7.29 | 0.664 | 0.500–0.779 | 0.083–0.907 | 7.63 | 5.82–11.85 |

Note. Congruence is the correlation between the original component loading vector and the matched, sign-aligned bootstrap loading vector. Bootstrap intervals are percentile-based.

**Supplementary Table S1B.** Loading-level bootstrap stability for brain-region loadings with |loading| ≥ 0.20.

| **Component** | **Region** | **Original loading** | **Bootstrap median** | **95% bootstrap interval** | **Same-sign resamples (%)** |
| --- | --- | --- | --- | --- | --- |
| PC1 | Brainstem | 0.403 | 0.370 | 0.160–0.471 | 100.0 |
| PC1 | Right Hippocampus | 0.369 | 0.332 | 0.140–0.426 | 99.9 |
| PC1 | Globus Pallidus | 0.363 | 0.337 | 0.083–0.451 | 99.7 |
| PC1 | Left Hippocampus | 0.349 | 0.317 | 0.096–0.411 | 99.7 |
| PC1 | Cerebellum | 0.304 | 0.289 | 0.014–0.408 | 98.2 |
| PC1 | Putamen | 0.298 | 0.262 | 0.064–0.389 | 99.0 |
| PC1 | Left Lateral Ventricle | -0.233 | -0.225 | -0.419–0.097 | 85.0 |
| PC1 | Occipital Gray Matter | -0.273 | -0.249 | -0.371–0.017 | 96.5 |
| PC2 | Right Lateral Ventricle | 0.373 | 0.342 | 0.096–0.436 | 99.7 |
| PC2 | Left Lateral Ventricle | 0.354 | 0.326 | 0.059–0.450 | 99.4 |
| PC2 | Third Ventricle | 0.337 | 0.295 | 0.128–0.385 | 99.9 |
| PC2 | Thalamus | 0.213 | 0.194 | -0.056–0.346 | 93.5 |
| PC2 | Amygdala | -0.228 | -0.207 | -0.353–0.015 | 95.5 |
| PC2 | Frontal Gray Matter | -0.291 | -0.260 | -0.378–-0.042 | 99.0 |
| PC2 | Parietal Gray Matter | -0.316 | -0.284 | -0.404–-0.027 | 98.2 |
| PC2 | Temporal Gray Matter | -0.372 | -0.320 | -0.422–-0.175 | 100.0 |
| PC3 | White Matter | 0.634 | 0.484 | 0.122–0.650 | 99.6 |
| PC3 | Thalamus | -0.253 | -0.214 | -0.491–0.158 | 88.2 |
| PC3 | Cerebellum | -0.284 | -0.199 | -0.460–0.113 | 91.2 |
| PC3 | Occipital Gray Matter | -0.331 | -0.280 | -0.488–0.064 | 94.8 |
| PC3 | Parietal Gray Matter | -0.400 | -0.321 | -0.525–-0.040 | 98.4 |
| PC4 | Putamen | 0.403 | 0.294 | -0.062–0.551 | 96.1 |
| PC4 | Fourth Ventricle | 0.396 | 0.288 | -0.112–0.587 | 92.5 |
| PC4 | Globus Pallidus | 0.338 | 0.255 | 0.013–0.425 | 98.0 |
| PC4 | Occipital Gray Matter | 0.270 | 0.172 | -0.145–0.462 | 84.5 |
| PC4 | White Matter | 0.222 | 0.247 | -0.233–0.560 | 78.8 |
| PC4 | Caudate | -0.232 | -0.184 | -0.532–0.327 | 79.7 |
| PC4 | Frontal Gray Matter | -0.237 | -0.203 | -0.473–0.214 | 86.7 |
| PC4 | Left Hippocampus | -0.246 | -0.169 | -0.448–0.126 | 86.2 |
| PC4 | Cerebellum | -0.293 | -0.256 | -0.476–0.083 | 93.0 |
| PC4 | Right Hippocampus | -0.303 | -0.207 | -0.433–0.017 | 96.2 |
| PC5 | Thalamus | 0.378 | 0.256 | -0.215–0.571 | 86.8 |
| PC5 | Occipital Gray Matter | 0.213 | 0.136 | -0.202–0.492 | 77.1 |
| PC5 | Left Lateral Ventricle | -0.251 | -0.161 | -0.376–0.098 | 88.2 |
| PC5 | Temporal Gray Matter | -0.312 | -0.209 | -0.487–0.210 | 84.9 |
| PC5 | Right Lateral Ventricle | -0.329 | -0.217 | -0.406–0.069 | 93.4 |
| PC5 | Caudate | -0.342 | -0.219 | -0.622–0.370 | 78.0 |
| PC5 | Fourth Ventricle | -0.387 | -0.289 | -0.624–0.240 | 86.9 |
| PC5 | Amygdala | -0.486 | -0.321 | -0.583–0.142 | 93.7 |

Note. Same-sign resamples indicate the percentage of bootstrap solutions in which the loading retained the direction of the original solution. Bootstrap stability reflects internal resampling stability and does not establish external reproducibility.

**Supplementary Table S2A.** Component-level bootstrap stability of the KINARM principal-component solution. N = 44; 2,000 bootstrap resamples.

| **Component** | **Original variance (%)** | **Median congruence** | **IQR** | **95% bootstrap interval** | **Bootstrap variance, median (%)** | **Bootstrap variance, 95% interval (%)** |
| --- | --- | --- | --- | --- | --- | --- |
| PC1 | 16.48 | 0.819 | 0.707–0.892 | 0.481–0.959 | 18.55 | 9.80–25.16 |
| PC2 | 15.11 | 0.771 | 0.678–0.847 | 0.434–0.925 | 16.77 | 11.69–21.76 |
| PC3 | 10.08 | 0.721 | 0.601–0.832 | 0.267–0.939 | 10.82 | 6.46–17.35 |
| PC4 | 7.79 | 0.698 | 0.601–0.793 | 0.309–0.903 | 8.68 | 6.31–13.12 |
| PC5 | 5.49 | 0.492 | 0.336–0.624 | 0.039–0.802 | 6.94 | 5.73–10.17 |

Note. Congruence is the correlation between the original loading vector and the matched, sign-aligned bootstrap loading vector. Bootstrap intervals are percentile-based. These estimates describe internal resampling stability only.

**Supplementary Table S2B.** Loading-level bootstrap stability for KINARM loadings with |loading| ≥ 0.20.

| **Component** | **Variable** | **Original loading** | **Bootstrap median** | **95% bootstrap interval** | **Same-sign resamples (%)** |
| --- | --- | --- | --- | --- | --- |
| PC1 | Object Hit: total objects hit | 0.221 | 0.188 | 0.035–0.253 | 99.0 |
| PC1 | Visually Guided Reaching: left-arm reaction time | -0.223 | -0.176 | -0.272–-0.007 | 97.8 |
| PC1 | Visually Guided Reaching: left-arm path length | -0.218 | -0.178 | -0.261–-0.036 | 98.5 |
| PC1 | Visually Guided Reaching: right-arm reaction time | -0.212 | -0.168 | -0.249–0.005 | 97.3 |
| PC1 | Visually Guided Reaching: right-arm movement time | -0.222 | -0.188 | -0.255–0.008 | 96.8 |
| PC1 | Visually Guided Reaching: left-arm task score, repeated block | -0.221 | -0.185 | -0.258–-0.006 | 97.8 |
| PC1 | Visually Guided Reaching: left-arm metric score, repeated block | -0.243 | -0.201 | -0.270–-0.023 | 98.2 |
| PC1 | Visually Guided Reaching: right-arm task score, repeated block | -0.271 | -0.228 | -0.290–-0.086 | 99.8 |
| PC1 | Visually Guided Reaching: right-arm metric score, repeated block | -0.271 | -0.228 | -0.290–-0.086 | 99.8 |
| PC2 | Ball on Bar Level 1: completion time | -0.318 | -0.252 | -0.307–-0.131 | 99.9 |
| PC2 | Ball on Bar Level 1: time ratio | -0.295 | -0.240 | -0.301–-0.103 | 99.8 |
| PC2 | Ball on Bar Level 1: dwell time | -0.305 | -0.240 | -0.293–-0.126 | 99.8 |
| PC2 | Ball on Bar Level 2: completion time | -0.322 | -0.255 | -0.310–-0.145 | 99.7 |
| PC2 | Ball on Bar Level 2: time ratio | -0.254 | -0.207 | -0.267–-0.095 | 99.7 |
| PC2 | Ball on Bar Level 2: dwell time | -0.315 | -0.249 | -0.303–-0.145 | 99.8 |
| PC2 | Ball on Bar Level 2: errors | -0.208 | -0.170 | -0.234–-0.058 | 99.2 |
| PC2 | Ball on Bar Level 3: completion time | -0.309 | -0.250 | -0.305–-0.133 | 99.8 |
| PC2 | Ball on Bar Level 3: task score | -0.278 | -0.231 | -0.295–-0.079 | 99.7 |
| PC2 | Ball on Bar Level 3: metric score | -0.233 | -0.197 | -0.263–-0.050 | 99.4 |
| PC3 | Object Hit: task score | 0.236 | 0.175 | -0.049–0.287 | 95.0 |
| PC3 | Object Hit and Avoid: task score | 0.329 | 0.240 | 0.047–0.350 | 99.2 |
| PC3 | Object Hit and Avoid: metric score | 0.264 | 0.183 | -0.025–0.326 | 96.0 |
| PC3 | Object Hit and Avoid: standardized score | 0.334 | 0.245 | 0.052–0.347 | 99.3 |
| PC3 | Object Hit and Avoid: right-hand speed | -0.218 | -0.163 | -0.279–0.053 | 93.5 |
| PC3 | Object Hit and Avoid: left-hand speed | -0.298 | -0.220 | -0.324–-0.033 | 98.5 |
| PC3 | Object Hit and Avoid: composite task score | 0.329 | 0.240 | 0.047–0.350 | 99.2 |
| PC3 | Object Hit and Avoid: composite metric score | 0.264 | 0.183 | -0.025–0.326 | 96.0 |
| PC4 | Visually Guided Reaching: posture speed, limb 1 | -0.256 | -0.167 | -0.320–0.054 | 93.3 |
| PC4 | Visually Guided Reaching: movement time, limb 1 | 0.278 | 0.177 | -0.033–0.322 | 95.3 |
| PC4 | Visually Guided Reaching: posture speed, limb 2 | 0.259 | 0.196 | -0.042–0.341 | 95.2 |
| PC4 | Visually Guided Reaching: path-length ratio, limb 2 | 0.219 | 0.169 | -0.075–0.335 | 91.8 |
| PC4 | Visually Guided Reaching: right-arm task score | 0.244 | 0.181 | -0.100–0.359 | 89.8 |
| PC4 | Visually Guided Reaching: left-arm posture speed | -0.276 | -0.189 | -0.314–0.003 | 97.3 |
| PC4 | Visually Guided Reaching: left-arm movement time | 0.256 | 0.179 | -0.021–0.311 | 95.9 |
| PC4 | Visually Guided Reaching: right-arm movement time | 0.254 | 0.183 | 0.010–0.304 | 98.2 |
| PC5 | Arm Position Matching: right-arm task score | -0.288 | -0.168 | -0.354–0.173 | 83.8 |
| PC5 | Arm Position Matching: right-arm metric score | -0.300 | -0.176 | -0.363–0.180 | 84.4 |
| PC5 | Ball on Bar: overall task score | -0.209 | -0.101 | -0.372–0.219 | 72.8 |
| PC5 | Ball on Bar: overall metric score | -0.237 | -0.116 | -0.370–0.201 | 74.7 |
| PC5 | Object Hit: right-hand hits | -0.214 | -0.129 | -0.336–0.155 | 81.7 |
| PC5 | Object Hit: task score | -0.206 | -0.128 | -0.318–0.156 | 82.8 |
| PC5 | Object Hit: metric score | -0.281 | -0.160 | -0.351–0.153 | 84.4 |
| PC5 | Object Hit and Avoid: processing rate | -0.246 | -0.142 | -0.337–0.124 | 84.2 |
| PC5 | Visually Guided Reaching: posture speed, limb 2 | -0.226 | -0.121 | -0.343–0.179 | 77.4 |
| PC5 | Visually Guided Reaching: right-arm task score | -0.260 | -0.140 | -0.373–0.212 | 77.3 |
| PC5 | Visually Guided Reaching: right-arm metric score | -0.299 | -0.157 | -0.383–0.194 | 81.1 |

Note. Same-sign resamples indicate the percentage of matched, sign-aligned bootstrap solutions in which the loading retained the direction of the original solution. Loading-level stability does not establish external reproducibility.

**Supplementary Table S2C.** KINARM loadings for the five retained principal components. N = 44. Only |loading| ≥ 0.20 are shown; blanks indicate loadings below the display threshold. Component signs are arbitrary.

| **PC1 (16.5%)** | **PC2 (15.1%)** | **PC3 (10.1%)** | **PC4 (7.8%)** | **PC5 (5.5%)** |
| --- | --- | --- | --- | --- |
| Visually Guided Reaching: right-arm task score, repeated block (-0.271) | Ball on Bar Level 2 (angle-dependent ball): completion time (-0.322) | Object Hit and Avoid: standardized score (0.334) | Visually Guided Reaching: movement time, limb 1 (0.278) | Arm Position Matching: right-arm metric score (-0.300) |
| Visually Guided Reaching: right-arm metric score, repeated block (-0.271) | Ball on Bar Level 1 (fixed ball): completion time (-0.318) | Object Hit and Avoid: task score (0.329) | Visually Guided Reaching: left-arm posture speed (-0.276) | Visually Guided Reaching: right-arm metric score (-0.299) |
| Visually Guided Reaching: left-arm metric score, repeated block (-0.243) | Ball on Bar Level 2 (angle-dependent ball): dwell time (-0.315) | Object Hit and Avoid: composite task score (0.329) | Visually Guided Reaching: posture speed, limb 2 (0.259) | Arm Position Matching: right-arm task score (-0.288) |
| Visually Guided Reaching: left-arm reaction time (-0.223) | Ball on Bar Level 3 (freely moving ball): completion time (-0.309) | Object Hit and Avoid: left-hand speed (-0.298) | Visually Guided Reaching: posture speed, limb 1 (-0.256) | Object Hit: metric score (-0.281) |
| Visually Guided Reaching: right-arm movement time (-0.222) | Ball on Bar Level 1 (fixed ball): dwell time (-0.305) | Object Hit and Avoid: metric score (0.264) | Visually Guided Reaching: left-arm movement time (0.256) | Visually Guided Reaching: right-arm task score (-0.260) |
| Object Hit: total objects hit (0.221) | Ball on Bar Level 1 (fixed ball): time ratio (-0.295) | Object Hit and Avoid: composite metric score (0.264) | Visually Guided Reaching: right-arm movement time (0.254) | Object Hit and Avoid: processing rate (-0.246) |
| Visually Guided Reaching: left-arm task score, repeated block (-0.221) | Ball on Bar Level 3 (freely moving ball): task score (-0.278) | Object Hit: task score (0.236) | Visually Guided Reaching: right-arm task score (0.244) | Ball on Bar: overall metric score (-0.237) |
| Visually Guided Reaching: left-arm path length (-0.218) | Ball on Bar Level 2 (angle-dependent ball): time ratio (-0.254) | Object Hit and Avoid: right-hand speed (-0.218) | Visually Guided Reaching: path-length ratio, limb 2 (0.219) | Visually Guided Reaching: posture speed, limb 2 (-0.226) |
| Visually Guided Reaching: right-arm reaction time (-0.212) | Ball on Bar Level 3 (freely moving ball): metric score (-0.233) |  |  | Object Hit: right-hand hits (-0.214) |
|  | Ball on Bar Level 2 (angle-dependent ball): errors (-0.208) |  |  | Ball on Bar: overall task score (-0.209) |
|  |  |  |  | Object Hit: task score (-0.206) |

**Supplementary Figure S1. Brain-region PCA scree plot.** Eigenvalues are shown for the brain-region principal component analysis derived from the 18 regional normative percentile measures. The dashed horizontal line denotes the Kaiser criterion eigenvalue threshold of 1.0, the dotted line shows the broken-stick reference values, and the dot-dash line shows the 95th percentile null eigenvalues from permutation-based parallel analysis. The first five brain principal components were retained for downstream modeling based on a combination of the elbow in the scree plot and the Kaiser criterion.

**Supplementary Figure S2. KINARM sensorimotor PCA scree plot.** Eigenvalues are shown for the KINARM sensorimotor PCA. The dashed horizontal line denotes the Kaiser criterion eigenvalue threshold of 1.0, the dotted line shows the broken-stick reference values, and the dot-dash line shows the 95th percentile null eigenvalues from permutation-based parallel analysis. The first five KINARM principal components were retained for downstream modeling based on the elbow in the scree plot. Component- and loading-level bootstrap stability results are shown in Supplementary Tables S2A and S2B, and original loadings are shown in Supplementary Table S2C.

**Supplementary Table S3.** Bayesian model coefficients for the association between brain structural principal components and athletic performance group (N=51).

| **Predictor** | **Log-odds coefficient (95% CrI)** | **Odds ratio (95% CrI)** | **Pr(direction)** |
| --- | --- | --- | --- |
| Brain PC1 | 0.53 (0.04 to 1.02) | 1.70 (1.04 to 2.78) | 0.98 |
| Brain PC2 | 0.32 (-0.15 to 0.81) | 1.38 (0.86 to 2.24) | 0.90 |
| Brain PC3 | 0.02 (-0.47 to 0.51) | 1.02 (0.63 to 1.66) | 0.54 |
| Brain PC4 | 0.00 (-0.48 to 0.50) | 1.00 (0.62 to 1.65) | 0.51 |
| Brain PC5 | -0.13 (-0.60 to 0.34) | 0.87 (0.55 to 1.40) | 0.71 |
| Sex: M | -0.51 (-1.36 to 0.36) | 0.60 (0.26 to 1.43) | 0.87 |

Note: Bayesian cumulative logit model predicting ordered performance group from Brain PC1-PC5 and sex. Odds ratios are shown for interpretability. Pr(direction) was evaluated using a prespecified descriptive threshold of 0.95 but was not adjusted for multiplicity and should not be interpreted as confirmatory evidence.

**Supplementary Table S4A.** Performance-rating sensitivity analyses.

| **Model/predictor** | **Coefficient (95% CrI)** | **OR (95% CrI)** | **Pr(direction)** |
| --- | --- | --- | --- |
| **Five-level ordered rating** | | | |
| Brain PC1 | 0.52 (0.05 to 1.00) | 1.69 (1.05 to 2.73) | 0.98 |
| Brain PC2 | 0.39 (-0.08 to 0.87) | 1.48 (0.93 to 2.39) | 0.95 |
| Brain PC3 | -0.08 (-0.51 to 0.37) | 0.93 (0.60 to 1.45) | 0.63 |
| Brain PC4 | -0.01 (-0.49 to 0.46) | 0.99 (0.61 to 1.58) | 0.52 |
| Brain PC5 | -0.10 (-0.57 to 0.38) | 0.91 (0.57 to 1.46) | 0.65 |
| Sex: M | -0.46 (-1.32 to 0.40) | 0.63 (0.27 to 1.49) | 0.85 |
| **High-performance binary rating** | | | |
| Brain PC1 | 0.52 (-0.06 to 1.12) | 1.68 (0.94 to 3.06) | 0.96 |
| Brain PC2 | 0.25 (-0.32 to 0.83) | 1.29 (0.73 to 2.30) | 0.81 |
| Brain PC3 | 0.28 (-0.30 to 0.90) | 1.32 (0.74 to 2.47) | 0.81 |
| Brain PC4 | -0.02 (-0.62 to 0.56) | 0.98 (0.54 to 1.75) | 0.52 |
| Brain PC5 | -0.39 (-0.98 to 0.19) | 0.67 (0.37 to 1.20) | 0.91 |
| Sex: M | -0.56 (-1.44 to 0.34) | 0.57 (0.24 to 1.40) | 0.89 |
| **Top-rating binary rating** | | | |
| Brain PC1 | 0.29 (-0.33 to 0.92) | 1.33 (0.72 to 2.50) | 0.82 |
| Brain PC2 | 0.35 (-0.25 to 0.97) | 1.41 (0.78 to 2.64) | 0.87 |
| Brain PC3 | 0.49 (-0.16 to 1.20) | 1.64 (0.86 to 3.31) | 0.93 |
| Brain PC4 | -0.31 (-0.97 to 0.30) | 0.73 (0.38 to 1.35) | 0.84 |
| Brain PC5 | -0.30 (-0.89 to 0.30) | 0.74 (0.41 to 1.35) | 0.83 |
| Sex: M | -0.37 (-1.27 to 0.53) | 0.69 (0.28 to 1.70) | 0.79 |

Note. CrI = credible interval; OR = odds ratio; Pr(direction) = posterior probability that the coefficient is in the reported direction. The 5-point expert rating was reverse-coded so higher values indicated higher performance. The high-performance model compared ratings 1-2 with ratings 3-5, and the top-rating model compared rating 1 with ratings 2-5. All models included Brain PC1-PC5 and sex. Intercepts and ordinal thresholds are omitted for readability. Sport-composition sensitivity analyses are reported in Supplementary Table S4B.

**Supplementary Table S4B. Sport-composition sensitivity analyses for the primary performance model.**

| **Model/predictor** | **Coefficient (95% CrI)** | **OR (95% CrI)** | **Pr(direction)** |
| --- | --- | --- | --- |
| **Hockey-adjusted primary model (known sport; N = 49)** | | | |
| Brain PC1 | 0.49 (-0.03 to 1.02) | 1.63 (0.97 to 2.76) | 0.97 |
| Brain PC2 | 0.29 (-0.21 to 0.78) | 1.33 (0.81 to 2.18) | 0.87 |
| Brain PC3 | -0.01 (-0.50 to 0.49) | 0.99 (0.61 to 1.64) | 0.51 |
| Brain PC4 | 0.09 (-0.42 to 0.61) | 1.10 (0.66 to 1.83) | 0.64 |
| Brain PC5 | -0.15 (-0.61 to 0.32) | 0.86 (0.54 to 1.38) | 0.73 |
| Sex: M | -0.54 (-1.39 to 0.31) | 0.58 (0.25 to 1.37) | 0.89 |
| Hockey | 0.33 (-0.48 to 1.11) | 1.39 (0.62 to 3.04) | 0.79 |
| **Non-hockey subset (N = 27)** | | | |
| Brain PC1 | 0.31 (-0.33 to 0.96) | 1.36 (0.72 to 2.60) | 0.82 |
| Brain PC2 | 0.22 (-0.41 to 0.85) | 1.24 (0.66 to 2.34) | 0.75 |
| Brain PC3 | -0.04 (-0.62 to 0.55) | 0.96 (0.54 to 1.73) | 0.56 |
| Brain PC4 | 0.10 (-0.54 to 0.74) | 1.11 (0.58 to 2.09) | 0.62 |
| Brain PC5 | 0.05 (-0.61 to 0.71) | 1.05 (0.54 to 2.04) | 0.55 |
| Sex: M | -0.49 (-1.40 to 0.40) | 0.61 (0.25 to 1.49) | 0.86 |

Note. All outcomes used the primary ordered three-level performance rating. The hockey-adjusted model included hockey status in addition to Brain PC1-PC5 and sex. The non-hockey model excluded hockey athletes. CrI = credible interval; OR = odds ratio; Pr(direction) is descriptive and not multiplicity-adjusted.

**Supplementary Table S5.** Bayesian model coefficients for associations between brain structural principal components and KINARM sensorimotor principal components (N=44).

| **Outcome** | **Predictor** | **Coefficient (95% CrI)** | **Pr(direction)** |
| --- | --- | --- | --- |
| KINARM PC1 | Brain PC1 | 0.47 (-0.19 to 1.12) | 0.92 |
| KINARM PC1 | Brain PC2 | -0.23 (-0.89 to 0.43) | 0.76 |
| KINARM PC1 | Brain PC3 | 0.36 (-0.30 to 1.01) | 0.86 |
| KINARM PC1 | Brain PC4 | 0.21 (-0.44 to 0.85) | 0.74 |
| KINARM PC1 | Brain PC5 | -0.01 (-0.66 to 0.64) | 0.51 |
| KINARM PC1 | Sex: M | -0.25 (-1.19 to 0.70) | 0.69 |
| KINARM PC2 | Brain PC1 | -0.42 (-1.04 to 0.22) | 0.90 |
| KINARM PC2 | Brain PC2 | 0.06 (-0.60 to 0.72) | 0.57 |
| KINARM PC2 | Brain PC3 | -0.31 (-0.93 to 0.32) | 0.84 |
| KINARM PC2 | Brain PC4 | -0.03 (-0.65 to 0.61) | 0.54 |
| KINARM PC2 | Brain PC5 | 0.22 (-0.43 to 0.87) | 0.75 |
| KINARM PC2 | Sex: M | -0.03 (-0.94 to 0.90) | 0.52 |
| KINARM PC3 | Brain PC1 | -0.21 (-0.76 to 0.36) | 0.78 |
| KINARM PC3 | Brain PC2 | -0.17 (-0.74 to 0.41) | 0.72 |
| KINARM PC3 | Brain PC3 | 0.57 (0.01 to 1.13) | 0.98 |
| KINARM PC3 | Brain PC4 | -0.01 (-0.57 to 0.55) | 0.52 |
| KINARM PC3 | Brain PC5 | 0.10 (-0.46 to 0.66) | 0.64 |
| KINARM PC3 | Sex: M | 0.10 (-0.79 to 0.97) | 0.59 |
| KINARM PC4 | Brain PC1 | -0.15 (-0.68 to 0.39) | 0.72 |
| KINARM PC4 | Brain PC2 | 0.05 (-0.50 to 0.60) | 0.58 |
| KINARM PC4 | Brain PC3 | 0.07 (-0.44 to 0.59) | 0.61 |
| KINARM PC4 | Brain PC4 | 0.08 (-0.46 to 0.61) | 0.63 |
| KINARM PC4 | Brain PC5 | 0.11 (-0.44 to 0.64) | 0.65 |
| KINARM PC4 | Sex: M | -0.28 (-1.17 to 0.62) | 0.73 |
| KINARM PC5 | Brain PC1 | -0.18 (-0.65 to 0.29) | 0.77 |
| KINARM PC5 | Brain PC2 | -0.20 (-0.68 to 0.28) | 0.79 |
| KINARM PC5 | Brain PC3 | -0.19 (-0.66 to 0.28) | 0.79 |
| KINARM PC5 | Brain PC4 | 0.12 (-0.33 to 0.58) | 0.70 |
| KINARM PC5 | Brain PC5 | 0.05 (-0.43 to 0.53) | 0.57 |
| KINARM PC5 | Sex: M | -0.24 (-1.05 to 0.60) | 0.71 |

Note. Separate Bayesian linear regressions predicting KINARM PC1-PC5 from Brain PC1-PC5 and sex. Coefficients are posterior regression estimates with 95% credible intervals. Pr(direction) was evaluated using a prespecified descriptive threshold of 0.95 but was not adjusted for multiplicity and should not be interpreted as confirmatory evidence.

**Supplementary Table S6. Bayesian model coefficients for the association between brain structural principal components and reported concussion history (N = 50).**

| **Predictor** | **Log-odds coefficient (95% CrI)** | **Odds ratio (95% CrI)** | **Pr(direction)** |
| --- | --- | --- | --- |
| Brain PC1 | 0.45 (-0.08 to 1.00) | 1.57 (0.92 to 2.71) | 0.95 |
| Brain PC2 | -0.49 (-1.03 to 0.04) | 0.61 (0.36 to 1.04) | 0.97 |
| Brain PC3 | 0.02 (-0.50 to 0.56) | 1.02 (0.60 to 1.76) | 0.52 |
| Brain PC4 | -0.35 (-0.90 to 0.19) | 0.70 (0.41 to 1.20) | 0.90 |
| Brain PC5 | -0.01 (-0.55 to 0.53) | 0.99 (0.58 to 1.70) | 0.51 |
| Sex: M | 0.32 (-0.52 to 1.20) | 1.38 (0.59 to 3.31) | 0.76 |

Note. Concussion history was defined as one or more reported concussions versus none; one participant lacked concussion data. Odds ratios greater than 1 indicate higher odds of concussion history. CrI = credible interval; Pr(direction) = posterior probability that the coefficient is in the reported direction. Pr(direction) was evaluated using a prespecified descriptive threshold of 0.95 but was not adjusted for multiplicity and should not be interpreted as confirmatory evidence. The emphasized Brain PC1 and PC2 credible intervals included the null and are interpreted as suggestive directional evidence.

**Supplementary Figure S3. Individual-level normative percentile heatmap.** Deidentified BrainKey age- and sex-referenced regional percentiles are shown for each athlete, ordered by performance rating. Values are centered at the reference-population median (50th percentile); red denotes higher and blue denotes lower percentiles relative to that median. This display presents the participant-level inputs underlying the brain PCA.

**Supplementary Figure S4. Regional distributions of normative brain-volume percentiles.** BrainKey age- and sex-referenced percentile values are shown by region and performance group. Points represent individual athletes, boxes summarize the distributions, and dashed lines mark the reference-population median (50th percentile).
